# A checklist for patient safety rounds at the care pathway level

**DOI:** 10.1093/intqhc/mzu019

**Published:** 2014-03-09

**Authors:** Cordula Wagner, Caroline A. Thompson, Onyebuchi A. Arah, Oliver Groene, Niek S. Klazinga, Maral Dersarkissian, Rosa Suñol, on behalf of the DUQuE Project Consortium, N Klazinga, DS Kringos, MJMH Lombarts, T Plochg, MA Lopez, M Secanell, R Sunol, P Vallejo, P Bartels, S Kristensen, P Michel, F Saillour-Glenisson, F Vlcek, M Car, S Jones, E Klaus, S Bottaro, P Garel, M Saluvan, C Bruneau, A Depaigne-Loth, C Shaw, A Hammer, O Ommen, H Pfaff, O Groene, D Botje, C Wagner, H Kutaj-Wasikowska, B Kutryba, A Escoval, A Lívio, M Eiras, M Franca, I Leite, F Almeman, H Kus, K Ozturk, R Mannion, OA Arah, M DerSarkissian, CA Thompson, A Wang, A Thompson

**Affiliations:** 1 NIVEL, Netherlands Institute for Health Services Research, Utrecht, The Netherlands; 2 Department of Public and Occupational Health, EMGO+ Institute for Health and Care Research, VU University Medical Center, Amsterdam, The Netherlands; 3 Department of Epidemiology, Fielding School of Public Health, University of California, Los Angeles (UCLA), Los Angeles, CA, USA; 4 Palo Alto Medical Foundation Research Institute, Palo Alto, CA, USA; 5 Center for Health Policy Research, University of California, Los Angeles, CA, USA; 6 Department of Health Services Research and Policy, London School of Hygiene & Tropical Medicine, London, UK; 7 Department of Public Health, Academic Medical Center, University of Amsterdam, Amsterdam, The Netherlands; 8 Avedis Donabedian University Institute, Autonomous University of Barcelona, Barcelona, Spain; 9 Red de Investigación en Servicios de Salud en Enfermedades Crónicas (REDISSEC), Barcelona, Spain

**Keywords:** quality improvement, quality management, external quality assessment, measurement of quality, surgery, professions, hospital care

## Abstract

**Objective:**

To define a checklist that can be used to assess the performance of a department and evaluate the implementation of quality management (QM) activities across departments or pathways in acute care hospitals.

**Design:**

We developed and tested a checklist for the assessment of QM activities at department level in a cross-sectional study using on-site visits by trained external auditors.

**Setting and participants:**

A sample of 292 hospital departments of 74 acute care hospitals across seven European countries. In every hospital, four departments for the conditions: acute myocardial infarction (AMI), stroke, hip fracture and deliveries participated.

**Main Outcome Measures:**

Four measures of QM activities were evaluated at care pathway level focusing on specialized expertise and responsibility (SER), evidence-based organization of pathways (EBOP), patient safety strategies and clinical review (CR).

**Results:**

Participating departments attained mean values on the various scales between 1.2 and 3.7. The theoretical range was 0–4. Three of the four QM measures are identical for the four conditions, whereas one scale (EBOP) has condition-specific items. Correlations showed that every factor was related, but also distinct, and added to the overall picture of QM at pathway level.

**Conclusion:**

The newly developed checklist can be used across various types of departments and pathways in acute care hospitals like AMI, deliveries, stroke and hip fracture. The anticipated users of the checklist are internal (e.g. peers within the hospital and hospital executive board) and external auditors (e.g. healthcare inspectorate, professional or patient organizations).

## Introduction

Executive or leadership walk rounds are widely used to improve patient safety but are also an activity studied on a limited basis. In a review of the literature, eight studies were found that evaluated walk rounds (executive or interdisciplinary), including one cluster-randomized trial. All studies reported improvements in (some domains of) safety culture and staff perceptions, but not on reduced safety risks or improved patient outcomes [1]. Leadership walk rounds vary between hospitals, but in general they consist of visits by members of the hospital executive board, senior leaders or risk managers to patient care areas to discuss patient safety issues with front-line care providers [2–4]. Mostly open-ended questions are used to discuss human error and specific safety risks, but not all rounding interventions use a structured format. To improve the effectiveness of these walk rounds, it may help to use a structured format with specific questions to evaluate the risks within a department and get the Plan-Do-Check-Act improvement cycle running. Feedback to involved unit caregivers about actions taken as a result of the walk rounds is essential to build trust and solve patient safety problems [4]. There is an indefinite number of possible actions to optimize and improve the care for individual patients. In general, professionals strive everyday for the best possible care for their patients, but limitations in human factors and organizational shortcomings sometimes hinder the quality of care delivered.

The aim of this study was to define a checklist that can be used to assess the implementation of quality management (QM) activities across four pathways in acute care hospitals. Based on the notion that QM can support quality improvement and reduce safety risks, we will focus on three areas, e.g. quality improvement covers quality policy and resources for improvement, evidence-based practice focuses on clinical guidelines and specific indicators, and patient safety strategies (PSS) covers activities and resources that can prevent harm to patients.

## Methods

### Setting and participants

The study took place in the context of the DUQuE project which ran from 2009 to 2013 [5, 6]. The data collection for this portion of the study took place in 74 hospitals visited by experienced external auditors in France, Poland, Turkey, Portugal, Spain, Germany and Czech Republic. The hospitals were randomly selected from a list of hospitals by the coordinator of the project. Eligibility criteria were as follows: acute care hospital, >130 beds and delivering care for the following four conditions, e.g. acute myocardial infarction (AMI), hip fracture, stroke and deliveries. In each participating hospital, the care processes of four care pathways were investigated. The conditions were chosen for their high financial volume, high prevalence, the different types of patients and specialists they cover, and the possibility of finding complications to have enough variance for the analysis in the sample. A checklist with specific questions for the site visits of the four care pathways were developed and used by trained external auditors from the respective countries. Ethical approval was obtained by the project coordinator at the Bioethics Committee of the Health Department of the Government of Catalonia (Spain).

### Measures: selection of questions for checklist

To decide on the content of each of the QM constructs (continuous quality improvement, evidence-based practice and PSS), we reviewed different sources. For quality improvement, we reviewed essential activities described in accreditation literature [7–12] and selected areas that were consistent across the different sources. For evidence-based management, we mapped the quality standards to evaluate compliance with clinical guidelines from the NICE (National Institute for Health and Care Excellence) [13, 14] and SIGN (Scottish Intercollegiate Guidelines Network) audit tools [15, 16], which are based on high evidence recommendations. Though each evidence-based measure was different for each condition, all include criteria related to admission, acute care, rehabilitation (if appropriate) and discharge.

For PSS, we mapped patients' safety recommendations, e.g. High fives, WHO programs and recommendations of the Patient's safety Alliance and Patient safety agencies and Required Organizational Practices (ROPs) from Canada accreditation [9]. The aim was to identify evidence-based practices that mitigate risk and contribute to improving the safety of health services. Final questions focused on identification, infection control, medication, life support, adverse events and security. We excluded questions about safety injections which are of global coverage in all countries where we performed the site visits.

A decision was taken early to use trigger questions that were appropriate across all four conditions of the study. In that sense, the process of selecting and developing trigger questions focused on generic and non-disease-specific measures for all domains except evidence-based management, questions for which were based on organizational guidelines for each specific condition. In all cases, we selected observable activities and documents in these areas to allow discussion and evaluation of QM and safety risks at the pathway level. The assumption is that the selected trigger questions can give a picture of the more general view of practices in a specific pathway. The final set of trigger questions consisted of 7 questions focusing on quality improvement, 9–14 questions on evidence-based practice, 12–14 questions on PSS and 2–4 questions about the organizational structure of the pathway. The number of questions differs across conditions because some questions were condition specific. The answers to the questions were evaluated by the auditor on a 0 to 4 compliance scale (0 = no or negligible compliance; 1 = low compliance; 2 = medium compliance; 3 = high, extensive compliance; 4 = full compliance) with the option of selecting ‘not applicable’ as appropriate. Explicit criteria were developed to rate the position for each item (final set of items can found in Table A1).

### Data collection

Data were collected during an external audit and through a checklist designed specifically for this project. Our criteria for this design aimed to: (i) minimize preparation time for the hospital, hence no self-assessment, (ii) limit staff interview time, thus focus on documentary evidence first and talk with staff later, (iii) avoid direct access to patients, or their personal records, (iv) require minimal analysis, interpretation or free text by auditors, (v) allow for documentation within 1 day by a team of two auditors and (vi) make it applicable to hospitals in all participating countries. The checklist for the audit process was piloted in two hospitals in different countries and translated into four languages (the other countries decided to use it in English). A data collection manual was developed. External auditors with previous experience in hospital accreditation and no relationship with the hospital in question conducted the visits to each hospital and each one of the selected departments. Every hospital and department were visited by a two-auditor team. A lead auditor for each country was centrally trained to unify the use of the checklist across participating countries. Training included theoretical and practical information, instructions on the main aspects to be assessed and scoring guidance. The lead auditor trained the second auditor. In total, 14 external auditors were gathering the data: 2 in each country. An IT platform was also developed for the audit tool to provide auditors with guidance to ensure homogeneity of data collection and provide continuous online support. The process took 1 day executed by two auditors, and no hospital professionals were made aware of audit contents beforehand. Data were collected between May 2011 and February 2012.

### Statistical analysis

Given that we gathered data in person using the auditors, we had no missing values for any items on the questionnaire. In total, complete data were available for 292 unique hospital departments that dealt with four conditions (namely, AMI, hip fracture, stroke and child deliveries). We began the analysis by describing characteristics of the sample of hospitals in each of the four pathways. Next, we aggregated items to develop four pathway-level quality measures, specialized expertise and responsibility (SER), evidence-based organization of pathways (EBOP), PSS and clinical review (CR). A score for each of these scales was computed by taking the mean of items used to build the respective scale. For each pathway, a specific analysis has been done. Exploratory factor analysis and theory guided our choice of items to aggregate for each scale. While exploratory factor analysis was used to reduce and determine which items would be aggregated to build a scale for (SER) and CR (Appendix 3), the items comprising EBOP and PSS were determined based on theoretical importance and background knowledge. It was not possible to build one generic scale for the EBOP, because of the different items across pathways. The other scales developed in this analysis used the same items to compute scores for each pathway. Despite the same items being used across pathways for the quality measure ‘patient safety strategies’, no generic scale for the four pathways revealed after factor analysis.

We provide pathway-specific means and standard deviations of each scale, and the mean and interquartile range of items that comprise the respective scales. We also report the percentage of observations in each pathway that had the lowest (or floor) and highest (or ceiling) values for each of the items. Lastly, we used Pearson's correlation coefficients to examine the relationship between the four pathway-level quality measures separately for each pathway. All analyses were conducted in SAS version 9.3 (SAS Institute, Inc., Cary, NC, USA).

## Results

Across the 7 countries, 74 randomly selected hospitals were visited to discuss and observe quality and safety procedures at 4 departments. Most departments were part of public hospitals with 501 to 1000 beds, and 44% were teaching hospitals. Background characteristics of the participating departments are given in Table 1.

**Table 1:** Characteristics of pathways by condition (*n* = 292)

Hospital characteristics	AMI, *n* = 72 (%)	Deliveries, *n* = 72 (%)	Hip fracture, *n* = 74 (%)	Stroke *n* = 74 (%)
Teaching status, *n* (%)				
Teaching	32 (44)	33 (46)	33 (45)	33 (45)
Non-teaching	40 (56)	39 (54)	41 (55)	41 (55)
Ownership, *n* (%)				
Public	59 (82)	58 (81)	59 (80)	59 (80)
Private (or mixed ownership)	13 (18)	14 (19)	15 (20)	15 (20)
Number of beds, *n* (%)				
<200	7 (10)	6 (8)	7 (9)	7 (9)
200–500	21 (29)	22 (31)	22 (30)	22 (30)
501–1000	30 (42)	31 (43)	31 (42)	31 (42)
>1000	14 (19)	13 (18)	14 (19)	14 (19)

In Table 2, the distribution of the four QM scales at department level is given. The seven items for quality improvement could be reduced by factor analysis to the three-item-scale CR. The questions on evidence-based practice could be split into the three-item-scale specialized expertise and responsibilities, and a sum score for EBOP.

**Table 2:** Distribution of scores for SER, EBOP, PSS and CR

Scale and items^a^	AMI (*n* = 72)		Deliveries (*n* = 72)		Hip fracture (*n* = 74)		Stroke (*n* = 74)	
	Average scores	SD	Average scores	SD	Average scores	SD	Average score	SD
SER	2.7	1.1	2.8	1.1	2.2	0.9	2.7	1.2
EBOP	3.2	0.9	3.7	0.3	2.3	1.0	3.0	1.0
PSS	2.6	0.5	2.7	0.6	2.5	0.5	2.5	0.6
CR	2.1	1.4	2.3	1.4	1.4	1.3	1.9	1.5

^a^Range of individual items and constructs: 0–4 (0 = no or negligible compliance, 1 = low compliance, 2 = medium compliance, 3 = high, extensive compliance, 4 = full compliance).

On a range of 0–4, the average score for specialized expertise and responsibilities lied between 2.2 and 2.8 for the different types of departments. The highest scores on the four scales are found for deliveries. In general, scores on EBOP were higher than those for CR. This pattern was consistent over the four types of departments.

In Table 3, the correlations between the four QM measures for the various types of departments are explored. The correlations for departments delivering care for AMI patients ranged for example from 0.25 (between ‘patient safety strategies’ and ‘evidence-based organization’) to 0.71 (between ‘evidence-based organization’ and ‘specialized expertise and responsibility’). For all other types of departments, each inter-measure correlation was below the pre-specified 0.70 threshold, deemed acceptable and showing the additional value of a measure [17]. A very strong correlation between the measures would mean that two scales measure, to a large extent, the same construct and one could be left out in the future. The results show that all four scales are an important part of QM at department level.

**Table 3:** Correlations between the four pathway (departmental)-level measures: SER, EBOP, PSS and CR

	AMI (*n* = 72)				Deliveries (*n* = 72)				Hip fracture (*n* = 74)				Stroke (*n* = 74)			
	SER	EBOP	PSS	CR	SER	EBOP	PSS	CR	SER	EBOP	PSS	CR	SER	EBOP	PSS	CR
SER	1				1				1				1.000			
EBOP	0.71	1			0.43	1			0.54	1			0.57	1		
PSS	0.31	0.25	1		0.44	0.14	1.000		0.24	0.19	1		0.16	0.20	1	
CR	0.47	0.40	0.36	1	0.55	0.40	0.42	1.000	0.17	−0.11	0.22	1	0.61	0.30	0.18	1

## Discussion

In this article, we described the development of a checklist for the assessment of QM activities at department level. We have used the checklist in four types of departments and across seven European countries. Based on the checklist, we could detect differences between departments in the implementation of SER, the way a department is organized (EBOP), the existing PSS and whether CR is used to give feedback to professionals about their performance. We also found differences in average scores on the scales between the four conditions. Three of the four scales are standardized and can be used across different types of departments. Only the scale EBOP is specific and different for every condition. The checklist is envisioned for internal use by professionals and (quality) managers in acute care settings and not directly for outpatient or long-term settings.

In the literature, various methods for the evaluation of performance in QM activities are described. All methods have strong and weak elements. Peer review usually focuses on physician performance, failing to assess systems in which care is delivered. Organizational peer-to-peer assessment to cross-share best practices, safety hazards, problems and actions that improve safety and organizational performance is an internally driven improvement method, but less independent and objective [18].

Auditing is considered to be an important activity of quality management systems (QMS). In many industrial disaster inquiries, the conclusion is that auditing of safety procedures and QMS was defective, and effectiveness of QMS is hindered by the inappropriate use of audit tools. Results of audits should be aligned with the Plan-Do-Check-Act cycle to achieve necessary improvements.

Criteria for clinical practice audits are useful for self-assessment and quality improvement. During an audit, the reviewer is asking ‘Do you have implemented the activity’, and ‘How well is’ an activity been done compared with the question ‘How well should’ it be done. Godwin (2001) has described 14 steps in a clinical practice audit but did not give structured format for specific pathways [19].

Interdisciplinary rounds combine a structured format for communication with a forum for regular interdisciplinary meetings. In a controlled trial, the effect of structured interdisciplinary rounds has been evaluated. The results showed a significant reduction in adjusted adverse events rates in a medical teaching unit [20].

Compared with these methods, our newly developed checklist covers a combination of questions with regard to organizational aspects, professional expertise, safety procedures and learning based on feedback about performance.

### Strength and limitations

The checklist has been used by trained external auditors with expertise in healthcare. Knowledge of healthcare processes is important for the evaluation of specific QM activities in hospitals. Evaluations during an audit or site-visit might be biased by the subjective judgment of the auditor. Ideally, an inter-rater reliability study gives more insight into the extent of agreement between auditors. In our study, it was not practically possible to conduct an inter-rater reliability study, which would have meant that two auditors from each country would have to visit hospitals in another country. To support reliable evaluations, the checklist contains mainly of questions asking for traceable documents, activities and results, and the audit process was done by two auditors together. In our study, seven countries were involved.

Furthermore, country variation exists, and therefore, we strived for generic objective activities on the checklist and no country-specific activities. There are other quality strategies we did not measure or ask for, but, we selected trigger questions based on years of audit experience and limited the length of the checklist. Self-selection bias with regard to better performing hospitals is possible. Despite the random selection process, only motivated hospitals will accept the invitation for participation.

### Practical implications

A key feature of our checklist is the detection of differences between departments and pathways. As we know that there are differences in patient outcomes across participating departments and pathways, we wanted to develop QM measures, which can possibly explain differences in patient outcomes. Patient safety and risk reduction is a major concern of healthcare organizations. Safety rounds are a promising method for internal and external use by hospital managers, hospital management boards, board of trustees or external auditors of the healthcare inspectorate. A standardized checklist supporting these safety rounds might improve the validity of the evaluation process. Based on the checklist, specific feedback can be given which makes it easier to start improvements.

## Conclusion

The newly developed checklist can be used across various types of departments and pathways in hospitals like AMI, deliveries, stroke and hip fracture. Three of the four QM measures are identical for the four conditions: specialized expertise, PSS and CR. The organization of the various pathways is different because of the different needs of patients. Therefore, specific questions were needed to evaluate the evidence-based organization of pathways. Further research is needed to investigate acceptability and feasibility of using the measures in routine hospital settings.

## Funding

The study, “Deepening our Understanding of Quality Improvement in Europe (DUQuE)” has received funding from the European Community's Seventh Framework Programme (FP7/2007–2013) under grant agreement n° 241822. Funding to pay the Open Access publication charges for this article was provided by European Community's Seventh Framework Programme (FP7/2007–2013) under grant agreement no. 241822.

## Appendix 1

### The DUQuE Project Consortium

Klazinga N, Kringos DS, Lombarts MJMH and Plochg T (Academic Medical Centre-AMC, University of Amsterdam, THE NETHERLANDS); Lopez MA, Secanell M, Sunol R and Vallejo P (Avedis Donabedian University Institute-Universitat Autónoma de Barcelona FAD. Red de investigación en servicios de salud en enfermedades crónicas REDISSEC, SPAIN); Bartels P and Kristensen S (Central Denmark Region & Center for Healthcare Improvements, Aalborg University, DENMARK); Michel P and Saillour-Glenisson F (Comité de la Coordination de l'Evaluation Clinique et de la Qualité en Aquitaine, FRANCE); Vlcek F (Czech Accreditation Committee, CZECH REPUBLIC); Car M, Jones S and Klaus E (Dr Foster Intelligence-DFI, UK); Bottaro S and Garel P (European Hospital and Healthcare Federation-HOPE, BELGIUM); Saluvan M (Hacettepe University, TURKEY); Bruneau C and Depaigne-Loth A (Haute Autorité de la Santé-HAS, FRANCE); Shaw C (University of New South Wales, Australia); Hammer A, Ommen O and Pfaff H (Institute of Medical Sociology, Health Services Research and Rehabilitation Science, University of Cologne-IMVR, GERMANY); Groene O (London School of Hygiene and Tropical Medicine, UK); Botje D and Wagner C (The Netherlands Institute for Health Services Research-NIVEL, THE NETHERLANDS); Kutaj-Wasikowska H and Kutryba B (Polish Society for Quality Promotion in Health Care-TPJ, POLAND); Escoval A and Lívio A (Portuguese Association for Hospital Development-APDH, PORTUGAL); Eiras M, Franca M and Leite I (Portuguese Society for Quality in Health Care-SPQS, PORTUGAL); Almeman F, Kus H and Ozturk K (Turkish Society for Quality Improvement in Healthcare-SKID, TURKEY); Mannion R (University of Birmingham, UK); Arah OA, DerSarkissian M, Thompson CA and Wang A (University of California, Los Angeles-UCLA, USA); Thompson A (University of Edinburgh, UK) Tables A2–A3.[Table MZU019TB4][Table MZU019TB5]

**Table A1 MZU019TB4:** Overview of items of the checklist for safety rounds for four clinical services: AMI, stroke, HIP fracture and deliveries

	AMI	Stroke	Hip fracture	Deliveries	Source	Clarification
**Items of SER of each pathway**
There is a strategic group within the hospital responsible for the overall clinical management.	X	X	X	X	Composition and function documented in protocols or other sources	The group has to coordinate all the path management. Rate 2 if it is an informal group or not documented; rate 4 if current clinical policy decisions are documented
There are clinical leaders with specialist training who are formally recognized as having principal responsibility for the overall clinical care.	X	X	X	X	Lead and deputy specialist doctors named when asking	Ask the names of who is responsible for the OVERALL coordination of the path management (in different departments)
Evidence-based clinical guidelines have been formally adopted and disseminated by the clinical staff for the management of patients.	X	X	X	X	Approved guidelines available	Rate 2 if guidelines exist but are not evidence-based, not consistent between teams, not formally adopted by strategic group; Rate 4 if guidelines are formally adopted and documented
**Items of EBOP of each pathway**						
There are written criteria and procedures for fast track admission and treatment of patients presenting with acute chest pain.	X				Procedures in emergency room	Rate 2 if not formally adopted or out of date
Arrangements ensure that eligible STEMI (S–T elevation myocardial infarction) patients can receive thrombolysis within 30 min after arrival at the hospital.	X				Procedures written for rapid decision and intervention	Rate 2 if arrangements say within 60 min
Immediate access is available at all times (24/7) to a specialist physician to determine whether coronary revascularization is appropriate.	X				On-call information or other evidence provided in emergency room	Rate 2 if limited to weekdays, or daytime; Rate 4 if 24 h a day, 7 days a week
Facilities area immediately available for performance and transport for emergency coronary angiography.	X				Procedures written for rapid decision and intervention	Rate 2 if it is accessible within 1 h but off-site; Rate 4 if it is accessible immediate, on-site
Facilities are immediately available for performance and transport for percutaneous coronary intervention	X				Procedures written for rapid decision and intervention	Rate 2 if it is accessible within 1 h but off-site; Rate 4 if it is accessible immediate, on-site
There is an agreed procedure for appropriate patients directly be transport for ambulance personnel to a stroke unit.		X			Procedures in stroke unit or emergency room	
Agreed procedures ensure that patients with suspected stroke are assessed for thrombolysis receiving, if clinically indicated.		X			Procedures in stroke unit or emergency room	
A thrombolysis service is available 7 days a week in the hospital or by formal arrangement elsewhere.		X			On-call information or other evidence provided in emergency room	Rate 2 if limited to weekdays, or daytime Rate 4 if 24 h a day, 7 days a week
Agreed procedures ensure that patients with acute stroke have their swallowing screened be a specially trained healthcare professional.		X			Approved guidelines available	
Protocols and procedures are available in order for patients to receive brain imaging within 1 h after arrival at the hospital.		X			Procedures written for rapid decision and intervention	
Protocols are in place to ensure if documented multidisciplinary goals are agreed within 5 days after admission to the hospital.		X			Approved guidelines available	
There is immediate access (1 h) to a specialist acute stroke unit (or area) for those with persisting neurological symptoms.		X			Procedures written for rapid decision and intervention	
The guidelines require that medical staff assess patients suspected of having a fractured hip within 1 h after arrival in the ED (or of the incident if already in the hospital).			X		Procedures written for rapid decision and intervention	
The guidelines require a multidisciplinary assessment plan and individual goals for rehabilitation to be documented within 24 h post-operatively.			X		Approved guidelines available	
Magnetic resonance imaging is immediately available if hip fracture is suspected despite negative plain X rays.			X			
The guideline requires that all patients presenting with a fragility (pathological) fracture are managed on a ward with routine access to acute orthogeriatric medical support.			X		Approved guidelines available	
Whenever clinically appropriate, surgery is performed within 48 h after admission.			X		Ask for 5 cases admitted at the time of visit (if surgery before 48 h count 1, if not count 0. Enter result 3/5 = 0.6	
Guidelines require that all patients undergoing hip fracture surgery receive antibiotic prophylaxis.			X		Approved guidelines available	
Guidelines require that, if the patient's overall medical condition allows, mobilization begins within 24 h post-operatively.			X		Procedure manual, approved guidelines	
A structured, accurate record of all events during the antenatal, childbirth and postnatal periods is maintained for every woman and child.				X		Rate 9 if by law babies have the same medical record as mother
All women, who have epidural analgesia or an operative delivery, have their pain assessed using a pain assessment tool approved by the hospital.				X		
There is prompt access to ultrasound facilities with trained staff.				X		Rate 2 if limited service (i.e. except evening, weekends); Rate 4 if 24/7
There is a procedure that guarantees that all women who are identified in the screening program as at risk of rhesus disease are properly managed.				X	Procedure manual	Rate 2 of informal procedure
Each woman receives one-to-one midwifery care during established labor and childbirth by a trained midwife.				X	Procedure manual	Rate 2 if limited service (i.e. except evening, weekends); Rate 4 if 24/7
Epidural analgesia is available at all times.				x	Procedure manual	Rate 2 if limited service (i.e. except evening, weekends); Rate 4 if 24/7
Adult intensive care facilities and specialist medical backup are available on-site.				X	Procedure manual	Rate 2 if limited service (i.e. except evening, weekends); Rate 4 if 24/7
Patient monitoring equipment and clinical expertise in its management are available within the obstetric unit.				X	Staffing arrangements, availability	Rate 2 if limited service (i.e. except evening, weekends); Rate 4 if 24/7
There is a system in place to ensure that anesthetic and theater services respond within 30 min to obstetric emergencies and expedite delivery in the event of maternal or fetal compromise.				X	Procedure manual	Rate 2 if limited service (i.e. except evening, weekends); Rate 4 if 24/7
All babies are clinically examined prior to discharge from hospital and/or within 72 h of birth, by a suitable qualified healthcare professional.				X	Procedure manual	Rate 2 if limited service (i.e. except evening, weekends); Rate 4 if 24/7
**Items of PSS of each pathway**						
Patients are identified by bracelet	X	X	X	X	Observe 10 patients	Calculate patient with bracelets/total patients (i.e. 6/10 = 0.6. Introduce 0.6
Safety boxes for disposal of injection devices are available in sufficient quantities for the number of injections administered	X	X	X	X	Disposal boxes available	Disposal boxes available, include having boxes with available space. Rate 2 if boxes are insufficient or overflowed
Promotional hand hygiene reminders are on display in the workplace	X	X	X	X	Posters or protocol clear and visible	Rate 2 if too few, or unclear; Rate 4 if clearly visible in most clinical areas
Staff are provided with a readily accessible alcohol-based hand rub at the point of patient care	X	X	X	X	Location of dispensers	Rate 2 if insufficient numbers, staff areas only; Rate 4 if fully operational within reach of all patient beds
There is no concentrated potassium chloride (KCl) stored in patient service areas	X	X	X	X	Direct observation	Not stored in general medication cabinet; Rate 2 if stored in separate cabinet with limited access by staff on ward; Rae 4 if all concentrated KCI removed from ward
Diagrammatic instructions for resuscitation are available in resuscitation areas	X	X	X	X	Posters or protocol clear and visible	Rate 22 if it is only visible in some areas
Each emergency ‘crash cart’ has a completed checklist of equipment and supplies	X	X	X	X	Checklist in the crash cart	Rate 4 if checklist completed by identified staff member at least daily if crash cart is not sealed
There is a system to report adverse events to patients	X	X	X	X	Evidence of an adverse events reporting system	Rate 0 if no notification system; Rate 1 if exists, Rate 2 if <10 events reported and 4 if >10 events reported
During 2010, CR included analysis of reported adverse events	X	X	X	X	Quantified analysis recorded in peer review minutes	Rate 2 if only quantification and no analysis or conclusions documented; Rate 4 if clear conclusions are documented in patients’ events review
**Items of CR of each pathway (CR)**						
During 2010, CR included analysis of routine clinical indicators on the management of the condition	X	X	X	X	Indicators recorded in peer review/group minutes or in the audit/review report	Indicators can exist without other guidelines evaluation
There is a multidisciplinary audit/review of practice against the guidelines	X	X	X	X	Peer review/group minutes or in the audit/review report	Rate 4 if it is dated on 2010 or 2011 (year before data collection)
Professionals participate or have direct feedback on results of audit/review of practice against guidelines	X	X	X	X	Peer review/group minutes, audit/review report or report sent to professionals	Rate 4 if almost all clinicians participate together in formal review or have direct feedback of results in 2010 or 2011

Response categories for all items are: (0) no or negligible compliance, (1) low compliance, (2) medium compliance, (3) high, extensive compliance, (4) full compliance, (9) non applicable.

X = question is part of the checklist for the specific clinical service.

**Table A2 MZU019TB5:** Specialized expertise and CR: item and scale characteristics, internal consistency reliability and corrected item-total correlations for AMI, deliveries, hip fractures and stroke pathways (*n* = 74 per condition)

Scale and items	Factor loadings				Cronbach's alpha				Corrected item-total correlation			
	AMI	Del	Hip	Stroke	AMI	Del	Hip	Stroke	AMI	Del	Hip	Stroke
**Specialized expertise and responsibility (SER)**					0.69	0.65	0.46	0.76				
A strategic group within the hospital is responsible for the overall clinical management	0.63	0.57	0.49	0.69					0.53	0.46	0.33	0.60
A clinical leader with specialist training is formally recognized as having principal responsibility for overall clinical care of patients	0.58	0.55	0.50	0.65					0.48	0.44	0.34	0.57
Evidence-based clinical guidelines have been formally adopted and disseminated by clinical staff	0.62	0.58	0.29	0.69					0.51	0.47	0.19	0.60
**Clinical Review (CR)**					0.86	0.86	0.76	0.84				
During 2010, CR included analysis of routine clinical indicators on the management of the condition	0.64	0.59	0.36	0.65					0.60	0.57	0.34	0.62
A multidisciplinary audit/review of practice against guidelines	0.91	0.94	0.89	0.91					0.83	0.89	0.70	0.82
Professionals participate or have direct feedback on results of audit/review of practice against guidelines	0.88	0.95	0.91	0.93					0.78	0.85	0.76	0.85
